# EFFECT OF EARLY-LIFE DISCRIMINATION IN PARENTING ON THE PHYSICAL AND MENTAL HEALTH OF MIDDLE-AGED AND OLDER ADULTS

**DOI:** 10.1093/geroni/igad104.3719

**Published:** 2023-12-21

**Authors:** Chao Guo, Lan Liu, Jie Liu

**Affiliations:** Peking University, Beijing, Beijing, China (People’s Republic); Peking University, Beijing, Beijing, China (People’s Republic); Peking University, Beijing, Beijing, China (People’s Republic)

## Abstract

This study aimed to explore the early-life discrimination in parenting on the physical and mental well-being of middle-aged and older adults, while also exploring the potential mediating role of early-life health. By combined data from the China Health and Retirement Longitudinal Study (CHARLS) in 2018 and related life history survey in 2014, 8204 females and 7305 males aged 45-84 were analyzed in this study. Logistic regression was employed to analyze the association between early-life discrimination in parenting and the incidence of chronic diseases as well as depression in middle-aged and older individuals. The KHB method was used to assess the mediating effect of early-life health in these connections. Regarding gender-based discrimination in parenting, parental preference for sons during early life was found escalated the probability of chronic diseases by 18% (OR=1.18, 95%CI: 1.01-1.38) and increased depression risk by 16% (OR=1.16, 95%CI: 1.03-1.31) among middle-aged and older females. In terms of sibling-related discrimination, parental inclination towards specific siblings heightened the likelihood of depression during middle and older years among females (OR=1.17, 95%CI: 1.03-1.34). However, these effects were not significant in males. The study further identified that early-life health partially mediated the influence of gender-based parental preferences on the mental well-being of middle-aged and older females, accounting for 8.603% of the overall effect. These findings underscored the potential for early-life discrimination within parenting to impact both the physical and mental health of middle-aged and older females by shaping their early-life health status, consequently resonating throughout their mental well-being in later life.

